# Reactive Force Field Molecular Dynamics Investigation of NH_3_ Generation Mechanism during Protein Pyrolysis Process

**DOI:** 10.3390/molecules29092016

**Published:** 2024-04-27

**Authors:** Shuai Guo, Yu Wang, Shujun Zhu, Hongwei Qu, Deng Zhao, Xingcan Li, Yan Zhao

**Affiliations:** 1 School of Energy and Power Engineering, Northeast Electric Power University, Jilin 132012, China; guoshuaidq@126.com (S.G.); a13341442483@126.com (Y.W.); quhongwei0928@126.com (H.Q.); xingcanli@neepu.edu.cn (X.L.); 2 Shanxi Key Laboratory of Coal Flexible Combustion and Thermal Conversion, Datong 037000, China; zhushujun@iet.cn; 3 College of Vehicles and Energy, Yanshan University, Qinhuangdao 066000, China; 4 Shenyang Academy of Environmental Sciences, Shenyang 110167, China

**Keywords:** pyrolysis, protein, ammonia, N migration

## Abstract

The mechanism of ammonia formation during the pyrolysis of proteins in biomass is currently unclear. To further investigate this issue, this study employed the AMS 2023.104 software to select proteins (actual proteins) as the model compounds and the amino acids contained within them (assembled amino acids) as the comparative models. ReaxFF molecular dynamics simulations were conducted to explore the nitrogen transformation and NH_3_ generation mechanisms in three-phase products (char, tar, and gas) during protein pyrolysis. The research results revealed several key findings. Regardless of whether the model compounds are actual proteins or assembled amino acids, NH_3_ is the primary nitrogen-containing product during pyrolysis. However, as the temperature rises to higher levels, such as 2000 K and 2500 K, the amount of NH_3_ decreases significantly in the later stages of pyrolysis, indicating that it is being converted into other nitrogen-bearing species, such as HCN and N_2_. Simultaneously, we also observed significant differences between the pyrolysis processes of actual proteins and assembled amino acids. Notably, at 2000 K, the amount of NH_3_ generated from the pyrolysis of assembled amino acids was twice that of actual proteins. This discrepancy mainly stems from the inherent structural differences between proteins and amino acids. In proteins, nitrogen is predominantly present in a network-like structure (NH-N), which shields it from direct external exposure, thus requiring more energy for nitrogen to participate in pyrolysis reactions, making it more difficult for NH_3_ to form. Conversely, assembled amino acids can release NH_3_ through a simpler deamination process, leading to a significant increase in NH_3_ production during their pyrolysis.

## 1. Introduction

Ammonia (NH_3_) is a widely employed substance for synthesizing nitrogen fertilizers, serving as a crucial nitrogen source for plants and promoting crop growth [1,2]. This plays a vital role in nourishing nearly half of the global population. The conventional method for NH_3_ synthesis predominantly relies on the Haber–Bosch (H-B) process. This process involves the reaction of nitrogen gas (N_2_) and hydrogen gas (H_2_) under high-temperature and high-pressure conditions (673~873 K, 20~30 MPa), consuming a significant amount of global energy (1–2%) [3,4,5,6]. Given these challenges, there is a substantial focus on researching environmentally friendly, energy-efficient, and sustainable alternatives for NH_3_ production.

One promising avenue is the extraction of NH_3_ from nitrogen-rich biomass [7,8], which may emerge as a potential sustainable source of nitrogen and hydrogen [9]. Pyrolysis, a clean and efficient thermochemical conversion technology, entails the high-temperature treatment of biomass. This process can effectively convert the nitrogen in biomass into char, tar, and gaseous nitrogen [10]. At present, Wang et al. [11] have discovered that the pyrolysis of microalgae can generate a substantial amount of NH_3_, and this NH_3_ can be recovered and reused as a fertilizer. Zhao et al. [12] have also successfully investigated the impact mechanism of cellulose pyrolysis by elucidating the transformation between functional groups at high temperatures, thereby further demonstrating the feasibility of biomass pyrolysis. The prospect of using nitrogen-rich biomass for NH_3_ production holds promise for two main reasons: (1) the acquisition of nitrogen-rich biomass is sustainable and cost-effective; and (2) compared to the traditional Haber–Bosch (H-B) process, NH_3_ synthesis can be conducted at atmospheric pressure without the need for introducing hydrogen, simplifying the process and reducing energy consumption.

Nitrogen in biomass primarily exists in the form of proteins (amino acids) [11]. Current research on NH_3_ generation from biomass primarily involves nitrogen-containing models of biomass, such as proteins and amino acids. Wang et al. [9] utilized nitrogen-rich microalgal biomass microalgae for NH_3_ production through pyrolysis, revealing that nitrogen in the microalgae raw material samples primarily exists in the form of proteins. They conducted pyrolysis experiments using the 17 amino acids contained in microalgae proteins, and the results indicated that, at a temperature of 800 °C, the NH_3_ yield reached its maximum, approaching 30%. Guo et al. [13], when investigating the impact of CaO on the formation of NO*_x_* precursors during the pyrolysis of sludge proteins, found that the NH_3_ yield reached its maximum at 400 °C. The production of NH_3_ was primarily attributed to the deamination of small-molecule amines generated by the decomposition of large-molecule proteins. Li et al. [14], through FTIR spectroscopy, studied the pyrolysis behavior of glycine and diglycine, finding that their pyrolysis mechanism is similar to the process of protein pyrolysis, with NH_3_ being the primary nitrogen-containing substance. Li et al. [15] used TG-FTIR spectroscopy to investigate the pyrolysis of phenylalanine and tyrosine, revealing NH_3_, HNCO, and HCN as the primary gaseous products. However, there is currently limited theoretical research on the formation of NH_3_ during protein pyrolysis. Therefore, it is necessary to explore the nitrogen conversion mechanism and NH_3_ production mechanism during protein pyrolysis.

In recent years, the application of molecular dynamics (MD) simulations based on the reactive force field (ReaxFF) has proven successful in modeling the combustion and pyrolysis characteristics of complex compounds such as coal [16], biomass [17], and charcoal [18,19]. This methodology has provided a deeper understanding of the reaction processes. Zheng et al. [17] employed molecular dynamics simulations to elucidate the initial reaction mechanisms of cellulose pyrolysis. Their research indicated that, compared to a larger model with 7572 atoms, a model with 17,664 atoms exhibited a closer evolutionary trend to the major pyrolysis products observed by Py-GC/MS, thus revealing crucial reaction pathways which are challenging to capture experimentally. Castro Marcano et al. [20] conducted a thorough investigation into the combustion of Illinois No. 6 coal char, consisting of 7458 atoms. The largest molecular model constructed in this study encompassed over 50,000 atoms, specifically composed of C_26860_H_20897_O_2502_N_412_S_330_, and was tailored for Illinois No. 6 coal [21,22]. The findings suggest that coupling the reactive force field with more realistic carbon molecular models can serve as a useful simulation approach to examine the intricate chemistry involved in structural transformations and chemical reactions during coal combustion. Additionally, Zheng et al. [23] utilized the ReaxFF-MD technique to delve deeper into the specific chemical reactions occurring during the pyrolysis of a Liulin coal model, comprising 28,351 atoms, marking it as the second coal model to be investigated using ReaxFF-MD. The results indicate that ReaxFF-MD simulations are instrumental in gaining profound insights into the chemical reactions occurring within complex molecular systems. These illustrative research examples further demonstrate the applicability of the ReaxFF-MD method in the field of macromolecular research.

In summary, the theoretical research on nitrogen transformation during protein pyrolysis is limited. Therefore, it is necessary to explore the mechanisms underlying the conversion of fuel nitrogen to NH_3_ during protein pyrolysis. To address this, we employed a method that allows for a detailed examination of nitrogen transfer reactions during protein pyrolysis at the molecular level—specifically, ReaxFF-MD simulation. We selected a protein with a nitrogen content of up to 17 wt% as a model compound to elucidate the evolution of the three-phase products and pathways of nitrogen conversion during pyrolysis. To further discuss the influence of the intrinsic structure of the protein on nitrogen conversion, we disassembled the protein into its assembled amino acid structures as a comparative model, aiming to comprehensively compare the impact of the intrinsic interaction of the protein on NH_3_ generation.

## 2. Results and Discussion

### 2.1. Proteins and Amino Acids Are Separately Thermally Decomposed

**Figure 1:**
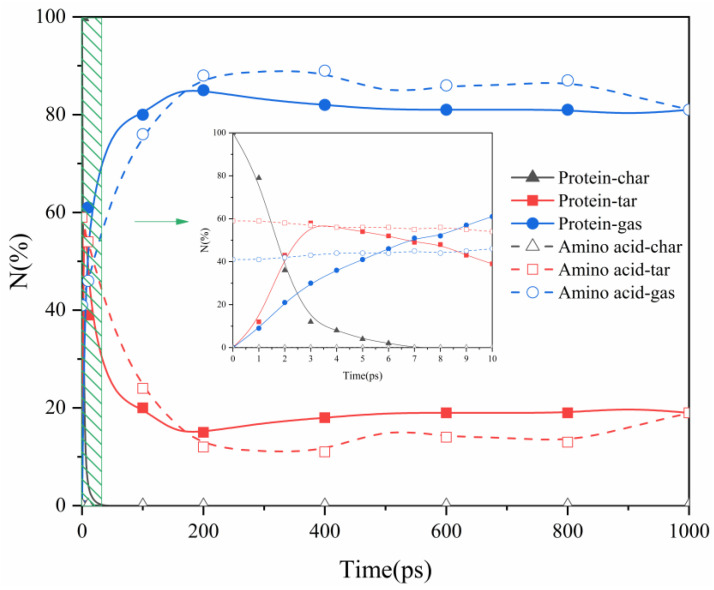
The distribution of three-phase products during the pyrolysis process of actual proteins and assembled amino acids at a temperature of 2000 K.

In previous studies on ReaxFF simulations [24], thermal decomposition products were categorized into three types—char, tar, and gas—based on the size of the carbon molecules, specifically, the C_40+_, C_5_-C_40_, and C_0_-C_4_ fragments. Taking 2000 K as an example, we investigated the distribution of nitrogen in char, tar, and gas fragments during the thermal decomposition of actual proteins and their assembled amino acids in the two simulation systems, as shown in Figure 1. In the actual thermal decomposition process of the proteins, the nitrogen content in char decreased from 100% to 0, whereas the nitrogen content in tar and gas gradually increased. Subsequently, the nitrogen content in the tar showed a decreasing trend, while gaseous nitrogen continued to increase. Ultimately, in the later stages of decomposition, the changes in the nitrogen content of the tar and gaseous nitrogen tended to stabilize. This is consistent with the findings of Chen et al. [25], who indicated that nitrogen migrates to smaller molecular fragments as large organic molecules decompose.

Based on the pyrolysis time, nitrogen migration during the protein pyrolysis process can be divided into two stages. In the initial stage, primary pyrolysis reactions take precedence, resulting in the gradual transfer of nitrogen from the unstable weak bonds within the large molecular structure of the protein to the gas and tar fragments. During the second stage, the predominant process is tar pyrolysis, leading to a substantial migration of nitrogen from tar to gas, concurrent with the ongoing pyrolysis reactions. This aligns with the observations made by Xu et al. [26], indicating that, during the pyrolysis of sludge and coal, nitrogen migrates to gases and tar fractions as larger molecules undergo decomposition. Consequently, in the pyrolysis of amino acids in our study, the primary nitrogen trends in char nitrogen, tar nitrogen, and gaseous nitrogen differed slightly from those of the proteins. This difference can be attributed to some limitations of the amino acid model in the ReaxFF-MD simulation, which did not consider changes in tar nitrogen. Therefore, in the pyrolysis of amino acids, the main nitrogen migration process included only the conversion of tar nitrogen into gaseous nitrogen, and no aggregation of amino acid molecules to produce peptides or their derivatives was observed. This observation is consistent with the findings of Leng et al. [27], who did not observe polymerization (dehydration) of glycine molecules during their molecular dynamics simulation study to produce peptides, DKP, or their derivatives. Clearly, through molecular dynamics simulations, utilizing actual proteins as models for pyrolysis research brings an investigation closer to the experimental conditions, rendering it more reliable.

In protein pyrolysis, two distinct stages can be discerned: the first stage, occurring between 0 and 7 ps, and the second stage, occurring after 7 ps. In our study, the first stage was short, indicating that large protein molecules rapidly decomposed into char at high temperatures. Moreover, the subsequent thermal decomposition rate of char was faster than the secondary thermal decomposition rate of tar nitrogen. In the second stage of protein pyrolysis, tar nitrogen decreased from 58% to 15% and was entirely converted into gaseous nitrogen. Similarly, in the pyrolysis process of assembled amino acids, from start to finish, tar nitrogen decreased from 59% to 11%, also entirely converting into gaseous nitrogen. These results suggest that, in both the actual proteins and in the amino acids they assemble, nitrogen primarily exists in the form of gaseous nitrogen during pyrolysis, with gaseous nitrogen being the main source of gaseous nitrogen [28].

### 2.2. The Influence of Pyrolysis Temperature

#### 2.2.1. The Comparative Impact of Pyrolysis Temperature on the Distribution of Three-Phase Products

**Figure 2:**
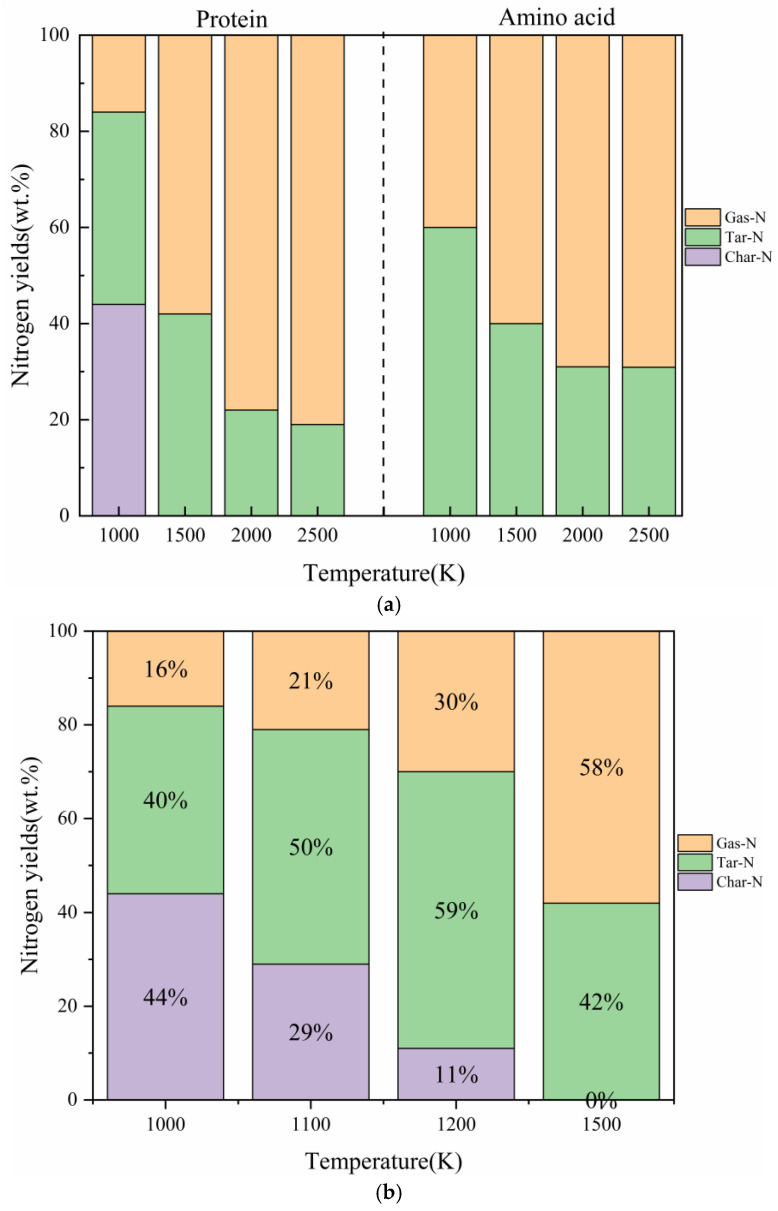
(**a**) Nitrogen distribution in the pyrolysis products of actual proteins and assembled amino acids. (**b**) Nitrogen distribution in the pyrolysis products of actual proteins.

Pyrolysis temperature plays a crucial role in determining the transformation and distribution of nitrogen. Figure 2a illustrates the impact of pyrolysis temperature on nitrogen distribution in the pyrolysis products of actual proteins and assembled amino acids. As the pyrolysis temperature increased from 1000 K to 2500 K, the tar nitrogen content in the pyrolysis products of the assembled amino acids significantly decreases, while the yield of gaseous nitrogen continued to increase. Specifically, the yield of gaseous nitrogen reached 40%, 60%, 69%, and 76% at temperatures of 1000 K, 1500 K, 2000 K, and 2500 K, respectively. This phenomenon can be explained by the fact that increasing the temperature favors the decomposition of tar nitrogen molecules into amino acids, causing them to enter the gaseous phase. As the temperature increased from 1000 K to 1500 K, the yield of char nitrogen in the actual proteins decreased significantly, dropping from 44% to 0. Tar nitrogen and gaseous nitrogen exhibited increasing trends. This can be explained by the higher temperature favoring the conversion of fuel nitrogen from char to volatile substances [29,30]. As the temperature continued to rise, reaching 2000 K and 2500 K, the yield of tar nitrogen began to decrease, dropping from 42% to 22% and 19%, respectively. This implies that, with increasing temperatures, the yield of tar nitrogen initially increased, releasing more nitrogen-containing substances in the form of large molecular volatiles. However, with a further temperature increase, more tar nitrogen underwent further decomposition into gaseous nitrogen products, resulting in a reduction in the yield of tar nitrogen [30]. In the temperature range of 1000~1500 K, the yield of gaseous nitrogen was relatively low but significantly increased at 2000 K. This can also be attributed to the secondary decomposition of nitrogen-containing substances in the volatiles.

When comparing the pyrolysis of actual proteins with that of the assembled amino acids, there was no discernible trend in char nitrogen in the pyrolysis products of the assembled amino acids in the temperature range of 1000~2500 K. This discrepancy contradicts the results of the experimental studies. This phenomenon explains the shortcomings of using amino acid models for molecular dynamics simulations. In agreement with the results of other experimental studies, the pyrolysis products of actual proteins exhibited a decreasing trend in char nitrogen. With increasing temperatures, char nitrogen gradually transformed into tar nitrogen and gaseous nitrogen. However, within the temperature range of 1000~1500 K, the yield of char nitrogen decreased directly from 44% to 0, and the changing trend in char nitrogen was not clearly evident. To validate the accuracy of the protein model, two additional temperature points, 1100 K and 1200 K, were added to this section within the temperature range of 1000~1500 K to determine the trend in the char nitrogen changes. As shown in Figure 2b, the yields of char nitrogen at temperatures of 1000 K, 1100 K, 1200 K, and 1500 K were 44%, 29%, 11%, and 0%, respectively. With an increase in temperature, the yield of char nitrogen significantly decreased, and the conversion of char nitrogen into tar nitrogen and gaseous nitrogen was evident. The changing trend in char nitrogen aligns with the experimental results of previous studies [9], further confirming the accuracy and feasibility of the protein model used in this study.

#### 2.2.2. The Impact of Pyrolysis Temperature on the Generation of NH_3_

**Figure 3:**
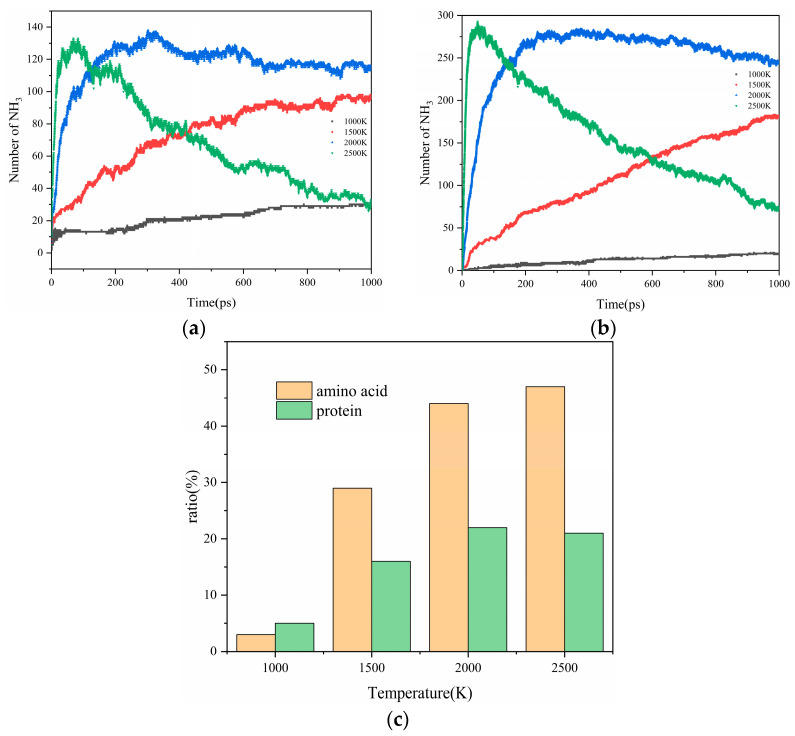
The quantity of NH_3_ produced during the thermal decomposition of actual protein (**a**) and assembled amino acids (**b**) changes over time. (**c**) The effect of temperature on the maximum conversion rate of the nitrogen in the proteins into NH_3._

NH_3_ is considered the primary pyrolysis gas [31]. Figure 3a,b illustrate the evolution of the evolving trends of NH_3_ over time at different temperatures during pyrolysis. The NH_3_ yield was defined as the maximum amount of NH_3_ in the pyrolysis products divided by the total nitrogen content. As the temperature increased from 1000 K to 1500 K, the quantity of NH_3_ increased over time. This can be attributed to the elevated temperature conditions promoting the secondary cracking of tar nitrogen, thereby releasing NH_3_. Nevertheless, as the temperature rose to 2000 K, there was an initial increase in the concentration of NH_3_, followed by a subsequent decline over time. Furthermore, at 2500 K, the decreasing trend became more pronounced. The substantial release of NH_3_ observed in the ascending trend may have originated from the decomposition of the unstable protein derivatives [32,33]. Regarding the descending trend, the literature [34] suggests that the variation in the quantity of NH_3_ produced from the pyrolysis of phenylalanine over time initially shows a slight increase, followed by a mild decrease. In our scenario, the significant reduction in NH_3_ quantity could be ascribed to intense interactions taking place under high-temperature conditions between NH_3_-N and other small molecular fragments produced from the pyrolysis of actual proteins or their assembled amino acids. This interaction accentuated the conversion of NH_3_-N into other nitrogen-containing small molecules. This implies that, during the pyrolysis process, NH_3_ not only existed as a product but also acted as a reactant in different reaction stages. At elevated temperatures, specifically 2000 K and 2500 K, the enhancing effect of the temperature on the consumption reaction of NH_3_ outweighed its positive impact on the generation reaction of NH_3_. Consequently, this led to a decreasing trend in net NH_3_ gas production over time.

During the thermal decomposition of proteins and their constituent amino acids, the maximum conversion rate of nitrogen into NH_3_ refers to the ratio of the maximum amount of NH_3_ produced to the total nitrogen content in the models. This metric enabled us to evaluate the impact of temperature on the selectivity of nitrogen conversion to NH_3_ in the two models. Figure 3c illustrates how the maximum conversion rate of nitrogen to NH_3_ varies with temperature for both models of proteins and their constituent amino acids. The maximum conversion rate of nitrogen to NH_3_ increased with temperature for both the actual proteins and the constituent amino acids, following a similar trend. However, there were significant quantitative differences. At a high temperature of 2000 K, the NH_3_ maximum conversion rates for the constituent amino acids and actual proteins reached 44% and 22%, respectively, indicating a high conversion efficiency. This disparity may have stemmed from the relatively simple amino structure of the constituent amino acids, which generally facilitates the deamination process [35]. In contrast, the nitrogen atoms in actual proteins are often embedded in complex networks, requiring the overcoming of higher energy barriers and decomposition into smaller molecules before the outermost nitrogen can be exposed and converted into NH_3_. This process is much more challenging than generating NH_3_ from its assembled amino acids. Given that both models exhibited high NH_3_ conversion rates at 2000 K and there was a relatively small decrease in NH_3_ production during the later stages of pyrolysis, we chose 2000 K as the representative temperature for our subsequent studies. This choice aimed to further explore the specific pathways of NH_3_ production from actual proteins and their constituent amino acids.

### 2.3. Comparison and Analysis of the NH_3_ Production Pathways for Actual Proteins and Their Assembled Amino Acids

**Figure 4:**
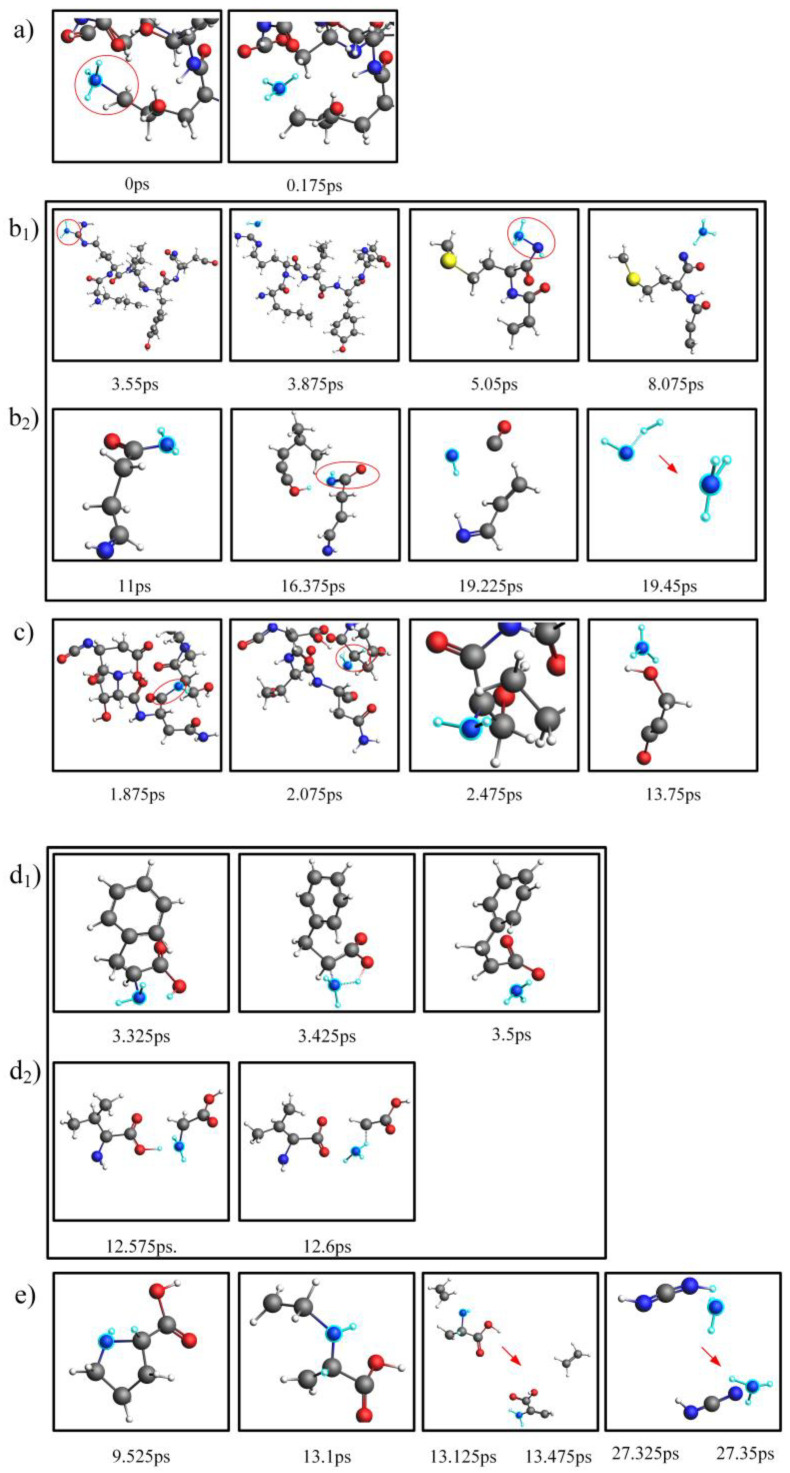
NH_3_ production pathways for actual proteins (**a**–**c**) and their assembled amino acids (**d_1_**–**e**).

In this section, we conducted a thorough analysis of the reaction networks during the pyrolysis process of actual proteins and their constituent amino acids, utilizing the advanced WF module of the AMS 2023.104software [36] and the ChemTraYzer 2.0 tool. The primary objective of this analysis was to explore the specific pathways leading to NH_3_ production in two distinct models at 2000 K. These findings have been comprehensively presented in Figure 4. The three forms of nitrogen present in proteins—NH_3_-N, NH_2_-N, and NH-N —are closely associated with three distinct pathways of NH_3_ formation, as illustrated in Figure 4a–c. Specifically, the NH_3_-N structure, naturally present at the edges of proteins, can directly detach to produce NH_3_, as depicted in Figure 4a. On the other hand, the NH_2_-N structure within protein molecules detaches its amino group at 3.875 ps and subsequently attaches to other small molecular structures produced during protein pyrolysis at 5.05 ps, combining with a H atom to form NH_3_ at 8.075 ps. This detailed process is shown in Figure 4b_1_. In another scenario, the H atom from the NH_2_ group is attracted by small molecular fragments generated during protein pyrolysis at 16.375 ps, converting the NH_2_ structure into an NH group. This free NH group then combines with H_2_ at 19.45 ps to produce NH_3_, as depicted in Figure 4b_2_. Given the complex macromolecular network structure of proteins, the NH groups located between carbon bonds are particularly abundant. These groups can form NH_3_ through a two-step hydrogenation process [37], as illustrated in Figure 4c. This discovery aligns with the research findings of Tan and Li [38,39,40], further confirming that the H radicals generated during pyrolysis can attack heterocyclic nitrogen to produce NH_3_.

In contrast, in assembled amino acids, the pathways of NH_3_ production are relatively simpler due to the presence of only two forms of nitrogen—NH_2_-N and NH-N—as shown in Figure 4d_1_–e. At 3.425 ps, the amino group on an amino acid can directly combine with a H atom to form NH_3_ at 3.5 ps, as depicted in Figure 4d_1_. Another pathway involves direct interactions between amino acids, with each providing an NH_2_ group and a H atom, ultimately leading to NH_3_ formation at 12.6 ps, as shown in Figure 4d_2_. Additionally, the NH groups in amino acids can also form NH_3_ through a two-step hydrogenation process, as illustrated in Figure 4e.

After examining the pathways of NH_3_ production in actual proteins and their assembled amino acids, it was observed that, in the actual proteins, the majority of NH_3_-N formation, specifically NH-N, was situated within their network structure. Overcoming the energy barrier of breaking down large molecular structures into smaller molecules was required to expose the nitrogen on the outermost layer. In contrast, the amino acids predominantly formed NH_3_-N through their inherent NH_2_-N structure, allowing direct deamination to form NH_3_. This was the primary reason why the proteins produced less NH_3_ than their assembled amino acids under the same conditions.

**Figure 5:**
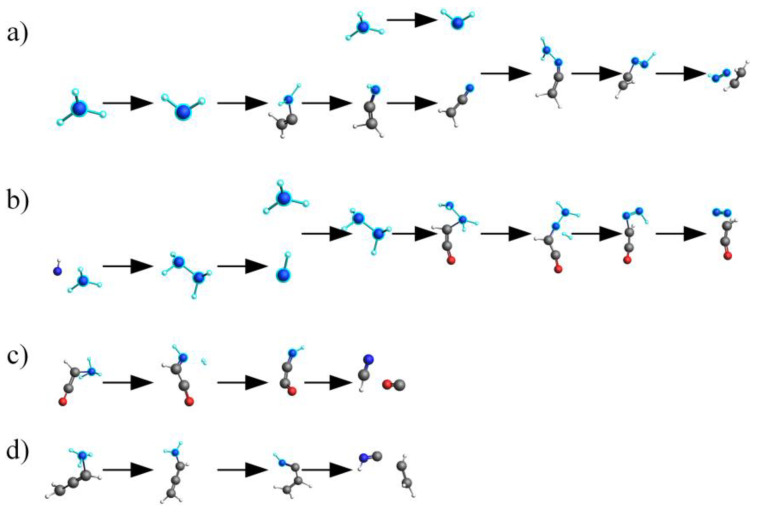
The pathway for the conversion of NH_3_, a thermal decomposition product of an actual protein, into N_2_ (**a**,**b**) and HCN (**c**,**d**).

Based on the above analysis, the pathways for NH_3_ production from the pyrolysis of assembled amino acids were similar to those of the actual proteins. However, due to the simple structure of amino acids, nitrogen is more easily converted into gaseous nitrogen during their thermal decomposition process [41], thereby contributing to an increased maximum conversion rate of nitrogen to NH_3_. This further confirmed the earlier finding that the NH_3_ gas maximum conversion rate was higher during the pyrolysis of amino acids. Therefore, a simple architecture improves selectivity for NH_3_. However, it is worth noting that, in the later stages of pyrolysis, for both the actual proteins and their assembled amino acids, NH_3_ served as both a product and a reactant. The analysis of the reconversion pathways of NH_3_ revealed that its decrease in quantity during the later stages of pyrolysis was primarily due to the action of H radicals, leading to the conversion of NH_3_ into HCN, N_2_, and other nitrogen-containing compounds. Because the NH_3_ reconversion pathways for both models were similar and NH_3_ had been converted into HCN and N_2_ in significant quantities, we used the NH_3_ reconversion pathway of the actual proteins as a representative and analyzed the pathways leading to the formation of HCN and N_2_, as shown in Figure 5. The pathways for the conversion of NH_3_ into N_2_ are illustrated in Figure 5a,b. In this process, the H on NH_3_ can be attracted by small molecules resulting from the actual protein or the breakdown of its assembled amino acids, forming NH*_i_* radicals. Subsequently, these NH*_i_* radicals are adsorbed onto nitrogen-containing small molecules during pyrolysis or interact directly with another NH_3_ molecule, progressively eliminating the H atoms around the nitrogen atom, ultimately forming N_2_. The pathway for the reconversion of NH_3_ into HCN is shown in Figure 5c,d. The H atoms on NH_3_ combine to form NH structures, which then detach in the form of H_2_. Alternatively, the surrounding H atoms are progressively attracted and adsorbed onto the carbon atoms of the pyrolysis’ small molecules, resulting in the detachment of HCN.

### 2.4. Mechanistic Analysis of the Actual Protein’s Generation of NH_3_

**Figure 6:**
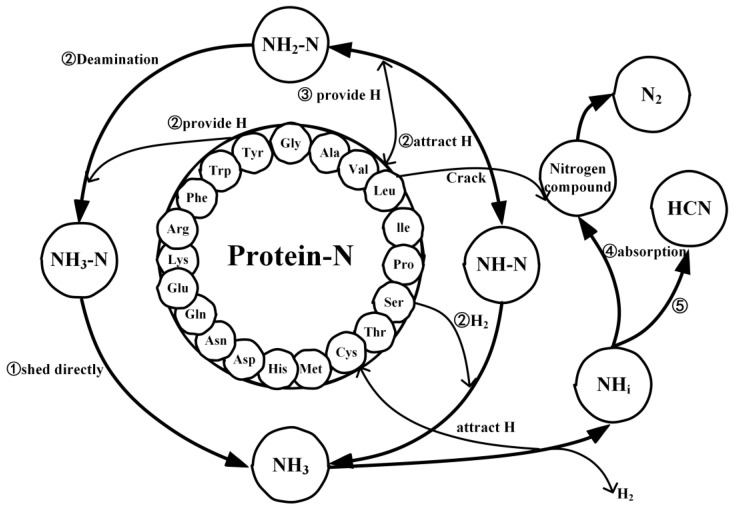
The mechanism diagram for the thermal decomposition of actual proteins and the generation of NH_3_.

Figure 6 details the reaction mechanism for the generation and transformation of NH_3_ during the pyrolysis process of macromolecular proteins at a temperature of 2000 K. Within proteins, nitrogen predominantly exists in three forms: NH_3_-N, NH_2_-N, and NH-N. The presence of hydrogen radicals significantly facilitates the conversion of these three nitrogen forms into NH_3_. The pathways for NH_3_ generation can be divided into three categories: direct shedding (Pathway ①); NH_2_-N attracts a hydrogen atom from small molecular fragments resulting from protein decomposition, or NH_2_-N loses a hydrogen atom to small molecular fragments, forming NH-N, which then combines with H_2_ (Pathway ②); and NH-N combines with hydrogen atoms from small molecular fragments twice (Pathway ③). However, in the later stages of the pyrolysis reaction, the amount of NH_3_ produced decreases over time. Through our reaction network analysis, it was found that this phenomenon is primarily due to the conversion of NH_3_ into N_2_ and HCN. Specifically, small molecular fragments from protein decomposition attract hydrogen atoms from NH_3_, or two hydrogen atoms from NH_3_ are directly shed to form H_2_, thereby converting NH_3_ into NH*_i_* radicals. Subsequently, NH*_i_* radicals combine with nitrogen atoms from nitrogen-containing compounds, resulting in the formation of N_2_ (Pathway ④), or they combine with carbon atoms from small molecular fragments to form HCN (Pathway ⑤).

## 3. Materials and Methods

In this study, proteins served as models for the pyrolysis of tobacco biomass sourced from Huamei Bio [42]. The preliminary preparation proceeded as follows: Initially, the original model consisted of 20 basic amino acids including 40 glycines, 43 alanines, 37 valines, 45 leucines, 28 isoleucines, 14 phenylalanines, 19 prolines, 7 tryptophans, 22 serines, 13 tyrosines, 10 cysteines, 16 methionines, 17 asparagines, 15 glutamines, 36 lysines, 30 aspartic acids, 32 glutamic acids, 35 arginines, 16 histidines, and 10 histamines, totaling 485 amino acids. The proportions of these amino acids to the total number of amino acids in the protein are presented in Table 1. Since hydrogen atoms were absent from the original model, we utilized the quantum chemical visualization software Gaussian View 5.0 to automatically add hydrogen to the selected model, ensuring the acquisition of a complete protein model. After successfully obtaining the complete model, in order to further investigate the pyrolysis simulation, we employed the ReaxFF-HE2 force field in the AMS software package [43,44,45] to carry out a precise and meticulous simulation analysis [46]. The potential energy function of the ReaxFF, given by (Equation (1)), encompassed terms for the total system energy, bond energy, penalty energy, valence angle energy, torsional angle energy, van der Waals energy, Coulomb energy, and bond-stretch energy. For more detailed information on ReaxFF, please refer to the literature [47].

(1) $$E_{\mathrm{System}}=E_{\mathrm{bond}}+E_{\mathrm{over}}+E_{\mathrm{angle}}+E_{\mathrm{tors}}+E_{\mathrm{vdWaals}}+E_{\mathrm{Coulomb}}+E_{\mathrm{Specific}}$$

**Figure 7:**
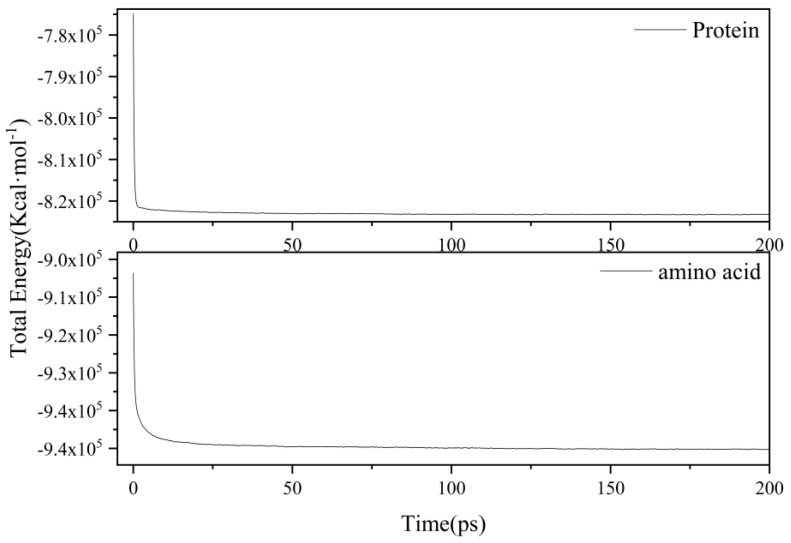
Energy distribution of the simulated system during the relaxation process.

The overall simulation process was as follows: Firstly, we imported the complete model file of the protein into the AMS software and constructed two separate systems. System 1 represented the protein model with a specific molecular formula, while System 2 consisted of 485 basic amino acids of 20 types (building blocks). Both systems were placed in a cubic box with a side length of 65Å, and their densities were set to 0.32 g/cm³ and 0.37 g/cm³, respectively. Subsequently, a relaxation process of 200 ps was performed at a temperature of 400 K to obtain an equilibrated initial structure. The energy distribution during this process is shown in Figure 7. After relaxation, using the NVT ensemble, high-temperature pyrolysis simulations were conducted on the final stable structures obtained after relaxation, at various target temperatures (1000 K, 1500 K, 2000 K, 2500 K). To precisely control the simulation temperature and delve deeper into its influence on the distribution of pyrolysis products, we selected a damping constant of 0.1 ps and the Berendsen thermostat. Regarding the setting of temperature, some literature [23] has adopted a programmed temperature increase method, which involves gradually heating the system from an equilibrium temperature to the desired simulation temperature at a specific heating rate. On the other hand, other literature [33] has chosen a constant temperature setting, which means setting the temperature directly to the target temperature without going through a heating process. In this study, we also adopted this method of isothermal pyrolysis. To accelerate the reaction kinetics, the simulation temperatures were set higher than the experimental temperatures to simulate sufficient reaction events within a shorter time frame [48]. During the high-temperature simulations, the time step was set to 0.25 fs, and the total simulation time was 1000 ps. This setup aided us in capturing the microkinetics of the pyrolysis reactions, providing a reliable simulation basis for a thorough analysis of system behavior. Finally, we utilized the WF module in the AMS software along with the ChemTraYzer 2.0 tool to analyze the reaction networks during the pyrolysis process of the protein and its constituent amino acids. This enabled a detailed investigation of the nitrogen transformation processes and NH_3_ production mechanisms in the pyrolysis of both systems.

## 4. Conclusions

In this study, a protein was chosen as a model for a molecular dynamics simulation. All the amino acid structures within the protein were utilized as comparative models to scrutinize the nitrogen conversion process during protein thermal decomposition in these two states. This study aimed to explore the impact of different interaction forms of amino acid structures within proteins on nitrogen transformation, ultimately revealing the mechanism of NH_3_ production during protein thermal decomposition. The conclusions are as follows:

1. Protein thermal decomposition occurs in two stages. The first stage involves the thermal rupture of unstable weak bonds in the protein’s large molecular structure, causing the gradual transfer of carbon and nitrogen to gas and tar fragments. The second stage is characterized by the thermal decomposition of tar, resulting in a notable migration of nitrogen from tar to the gas phase during the decomposition reaction.
2. In our study, the actual protein and its assembled amino acids exhibited significant temperature-dependent variations in their maximum conversion rate during thermal decomposition. Specifically, at 1000 K, 1500 K, 2000 K, and 2500 K, the maximum conversion rates of the actual protein were 5%, 16%, 22%, and 21%, respectively. In contrast, the maximum conversion rate from the thermal decomposition of the assembled amino acids was relatively high, measuring 3%, 29%, 44%, and 47% at the corresponding temperatures. This phenomenon can be explained by the fact that the actual protein must overcome an energy barrier to break weak bonds, thereby exposing nitrogen and forming NH_3_. This process is more challenging than the direct deamination of the assembled amino acids to produce NH_3_.
3. Under conditions of 2000 K, the formation of NH_3_ was primarily influenced by hydrogen radicals, causing the conversion of the nitrogen in the protein (NH_3_-N, NH_2_-N, NH-N) into NH_3_. Furthermore, the decrease in the quantity of NH_3_ in the later stages of thermal decomposition was attributed to its reconversion as a reactant, predominantly forming HCN and N_2_.

During the exploration process, the yield of NH_3_ generated from the thermal decomposition of the actual proteins was relatively low. To enhance the maximum conversion rate, we plan to increase the selectivity of nitrogen conversion to NH_3_ in proteins by introducing a hydrogen atmosphere or catalysts in the future. The aim of this study is to provide a theoretical reference for the generation of NH_3_ during the thermal decomposition of proteins in biomass.

## Figures and Tables

**Table 1:** Content of amino acids in the protein.

Amino Acids	Ratio%
Glycine	8.25
Alanine	8.87
Valine	7.63
Leucine	9.28
Isoleucine	5.77
Phenylalanine	2.89
Proline	3.92
Tryptophan	1.44
Serine	4.54
Tyrosine	2.68
Cysteine	2.06
Methionine	3.30
Asparagine	3.51
Glutamine	3.09
Threonine	7.42
Aspartic acid	6.19
Glutamic acid	6.60
Lysine	7.22
Arginine	3.30
Histidine	2.06
∑	100

## Data Availability

Data are contained within the article.
